# Predicting Risky and Aggressive Driving Behavior among Taxi Drivers: Do Spatio-Temporal Attributes Matter?

**DOI:** 10.3390/ijerph17113937

**Published:** 2020-06-02

**Authors:** Muhammad Zahid, Yangzhou Chen, Sikandar Khan, Arshad Jamal, Muhammad Ijaz, Tufail Ahmed

**Affiliations:** 1 College of Metropolitan Transportation, Beijing University of Technology, Beijing 100124, China; zahid@emails.bjut.edu.cn; 2 College of Artificial Intelligence and Automation, Beijing University of Technology, Beijing 100124, China; yzchen@bjut.edu.cn; 3 Department of Mechanical Engineering, King Fahd University of Petroleum & Minerals, KFUPM Box 5069, Dhahran 31261, Saudi Arabia; 4 Department of Civil and Environmental Engineering, King Fahd University of Petroleum & Minerals, KFUPM Box 5055, Dhahran 31261, Saudi Arabia; arshad.jamal@kfupm.edu.sa; 5 School of Transportation and Logistics, Southwest Jiaotong University, Chengdu 610031, China; m.ijaz58@yahoo.com; 6 UHasselt, Transportation Research Institute (IMOB), Agoralaan, 3590 Diepenbeek, Belgium; tufail.ahmed@uhasselt.be

**Keywords:** aggressive driving, traffic violations, hotspot analysis, Geographic Information System (GIS), machine learning, taxi drivers

## Abstract

Risky and aggressive driving maneuvers are considered a significant indicator for traffic accident occurrence as well as they aggravate their severity. Traffic violations caused by such uncivilized driving behavior is a global issue. Studies in existing literature have used statistical analysis methods to explore key contributing factors toward aggressive driving and traffic violations. However, such methods are unable to capture latent correlations among predictor variables, and they also suffer from low prediction accuracies. This study aimed to comprehensively investigate different traffic violations using spatial analysis and machine learning methods in the city of Luzhou, China. Violations committed by taxi drivers are the focus of the current study since they constitute a significant proportion of total violations reported in the city. Georeferenced violation data for the year 2016 was obtained from the traffic police department. Detailed descriptive analysis is presented to summarize key statistics about various violation types. Results revealed that over-speeding was the most prevalent violation type observed in the study area. Frequency-based nearest neighborhood cluster methods in Arc map Geographic Information System (GIS) were used to develop hotspot maps for different violation types that are vital for prioritizing and conducting treatment alternatives efficiently. Finally, different machine learning (ML) methods, including decision tree, AdaBoost with a base estimator decision tree, and stack model, were employed to predict and classify each violation type. The proposed methods were compared based on different evaluation metrics like accuracy, F-1 measure, specificity, and log loss. Prediction results demonstrated the adequacy and robustness of proposed machine learning (ML) methods. However, a detailed comparative analysis showed that the stack model outperformed other models in terms of proposed evaluation metrics.

## 1. Introduction

Aggressive driving behavior pose a major social and public health concern in urban metropolitans worldwide. During such situation, drivers commit or tend to commit combination of traffic violations in such a way that endangers other individuals or public property. Traffic violations can be categorized as aggressive or ordinary [1]. The aggressive ones involve drivers’ explicitly acting aggressively, whereas the ordinary ones consist of deliberately violating the traffic regulations without expressing aggressive motives. Risky and aggressive driving behavior of drivers is regarded as the one of the few leading cause of road traffic accidents (RTAs), particularly, in the People’s Republic of China (PRC) [2]. PRC has witnessed a tremendous industrial revolution during the past four decades. Motorization and auto-ownership have also increased exponentially, and so as the rate of RTAs. In PRC, a significant proportion of road traffic injuries (RTIs) are caused by traffic violations mainly associated with such uncivilized driving behaviour. PRC has a RTA fatality index value of 18.2 (per 100,000 persons) which is significantly higher compared to developed countries like the US (10.6) and the UK (5.2), and is also marginally high than neighboring developing countries. Similarly, the proportion of vulnerable road users (VRUs) who died on national roads has also rapidly increased from 52% in 2008 to 60% in 2016, in contrast to 22% of corresponding total road fatalities in the US [3,4]. According to a study by the Ministry of Public Security’s Transport Administration in China, there were a total of 50,400 road truck accidents throughout China in 2016, with a death toll of 25,000 and 46,800 injuries. A total of 17,242 taxi violations, including illegal parking violations and red-light running, occurred in Beijing within just one month in 2003, according to a report on traffic violations. It showed that fines were imposed on 27% of taxi drivers [5]. In 2015, the economic losses due to RTCs in PRC were estimated to be worth 1.08 billion yuan [6]. Such huge socio-economic burden could be substantially mitigated by adopting appropriate measures to discourage aggressive driving behaviour causing risky violations.

Previous studies suggest that aggressive driving attitudes and resulting RTCs events are the consequence of several interacting factors, including; driver attributes, vehicle characteristics, poor roadway design, and features, built environment, weather, and visibility conditions [7,8,9]. Driver related factors accounts for more than 90% of crash occurrences [10,11,12]. Some of the prevailing driver factors in this regard are: distractions (mainly because of mobile phone use), drunk driving, driving under fatigue, risky and aggressive driving attitudes that leads to various traffic violations i.e., over-speeding, red-light crossing, non-compliance with pedestrian signals, road markings, etc. Among all, traffic violations are reported to have a strong bearing on crash occurrences as well as associated crash severities [13]. Despite legislation and enforcement, risky driving and committing traffic violations continue to happen almost everywhere, and there has been no concrete solution to avoid them wholly. A recent World Health Organization (WHO) report suggests that various traffic rules and regulations have been legislated and enforced in true spirit only across 35 different countries around the world [14].

Studies suggest that traffic violations resulting from aggressive driving attitudes are mainly associated with variables such as socio-demographic attributes of drivers, seasonal variations, the period of day (peak or off-peak), type of highway, and characteristics of the built environment [15,16]. Interestingly, existing literature reports that taxi drivers in particular are more frequent violators than others [17]. Recently, several studies have attempted to explore factors contributing to risky and aggressive driving attitudes by taxi drivers, and their connection to RTCs [18,19]. Taxis constitute a vital component of urban road transport. Taxi drivers tend to have aggressive driving behaviour by violating traffic rules, as they seek to pick and drop the riders in a hurry to increase financial benefits. The conditions of road traffic and the number of prospective roadside passengers varies at different times of the day, which may influence the taxi drivers to commit a traffic violation. Taxi drivers are expected to travel between the designated lane markings. Abrupt lane changes could cause severe disturbance to traffic and are the primary source of a significant proportion of inter-city car accidents. Adequate road infrastructure is essential to ensure a smooth flow of traffic in the respective right of way. For example, urban streets need adequate, clear, and visible road markings and other traffic control devices (TCD). Proactive traffic control and forecasting could also help to enforce traffic rule regulations and discourage aggressive driving on urban streets [20,21,22]. Additionally, the presence of an appropriate number of trained police officers in a given location is expected to decrease the frequency of traffic violations significantly.

Studies have shown that the traffic violations caused by aggressive taxi driving are admissible evidence for crash indicators in urban metropolitans [23]. Thus, understanding the factors underlying traffic violations (caused by aggressive driving attitudes) made by taxi drivers is worth exploring. Although few studies conducted in past have examined several noticeable factors for traffic violations among taxi drivers, investigations on spatio-temporal factors are scarce. In this study, we analyzed patterns of various traffic violations made by taxi drivers (i.e., red-light violation, over-speeding, wrong-way driving, illegal parking, driving on dedicated lanes, and violation pedestrian signals and road markings) along general urban road in the city of Luzhou, China. The main contributions of the study are as follow:

- We used frequency-based nearest neighborhood cluster methods in Arc map Geographic Information System (GIS) to develop hotspots maps for different violation types that are vital for prioritizing and conducting treatment alternatives efficiently.
- Secondly, we predict and classify the occurrence of violations by taxi drivers using stack generalization technique. To the best of our knowledge, it has not been previously used in traffic violations prediction and classification.
- To demonstrate the efficacy of the proposed technique, a detailed comparison has been made with base models (AdaBoost and decision tree (DT)). The results demonstrate that the stack model outperformed the base models.

The rest of the paper is structured as follows. Section 2 reviews previous work regarding traffic violation prediction and classification. Section 3 presents the study area and data collection. Section 4 discusses the analysis of descriptive statistics and hotspots. Section 5 provides the fundamentals of proposed methods i.e., the decision tree (DT) model, the AdaBoost model, and the stack model in the context of the current study. Section 6 highlights results and discussions, model’s comparisons, and proposed mitigation strategies to counter aggressive driving. Finally, Section 7 summarizes key study findings, recommendations, and outlook for future studies.

## 2. Related Work

Aggressive driving attitudes and driving violations and their relationship to RTCs have been the focus of leading research in the public health domain, particularly during the last two decades. Numerous research studies have indicated that taxi drivers frequently engage in illegal maneuvers to make U-turns and reverse directions without catering for adjacent vehicles [2,24]. Traffic simulation have also proven to be very useful for analyzing the detailed driving behaviour in compliance to traffic regulations [25]. In addition, previous research has also focused on challenges and issues related to taxi drivers from multiple perspectives, such as odds of being involved in crashes [26,27,28], fatigue driving [29,30], vision problems [30,31], law compliance [17,32], risk taking [16,33], risk perception [34], and seat belt use [35]. Recently, studies have shown that traffic violations among taxi drivers mostly happened due to lack of correspondence among taxi drivers and law enforcement officers, job experience [17], age, education level [36], the existence of the complaint system, and economic pressure [16,36].

Research from earlier studies suggests that over-speeding is one of the most prevailing traffic violations encountered across different countries [37,38], which has resulted in a large number of severe and fatal crashes [39]. Furthermore, numerous studies show that the violation of speed not only increases the risk of fatal traffic accidents, but also makes them worse [40,41,42,43,44,45]. However, another potential factor impacting the rate of violation is the level of education. The survey showed that the majority of taxi drivers had junior or senior high school diplomas, and therefore only 1.8% had university or high school qualifications [46]. In addition, drivers with poor, educated backgrounds were more likely to be involved in risky driving practices than drivers with high educational background [47], which means that taxi drivers are more likely to have a higher violation rate. Likewise, other research in Turkey showed that taxi drivers who had previous crash encounters did not change their behaviour because they simply relate the cause of the accident to fate or misfortune rather to themselves [38]. Similarly, another study indicated that Malaysian airport limousine taxi drivers were found to be more aggressive and to confess traffic violations regarding speeding particularly during weekdays [48]. A detailed study was conducted by Sagberg and Ingebrigtsen to analyze and review patterns of aggressive driving trends among Norwegian drivers using driving incidents collected in preceding three year period [49].

In previous studies, several methods have been proposed to understand the relation between risky and aggressive driving with contributing factors. The relation between the levels of welfare and driving violations was analyzed by using the logistic regression method, and the data were collected from Urmia transport police and through a questionnaire. They concluded in the end that a meaningful relationship exists between the welfare level and failing to pay attention to police rules and regulations [50]. Wu et al. [32] used logistic regression to study the gaps between taxi drivers driving and novice drivers behind red-light in China using simulation methods. The results have shown that nonprofessional drivers are more likely to avoid crossing the red-light than taxi drivers. Meanwhile, taxi drivers also have a lower accident rate than inexperienced drivers. The study also performed using multinomial regression, examined driving violations, and subsequently, the risk of accidents involving novice Australian drivers. They found that inexperienced drivers subjecting driving violations are more likely to suffer accidents than more experienced drivers [51].

Additionally, the researchers tried to combine aggressive driving violations with a crash risk in a robust way. Three types of violations, including illegal overtaking, tailgating, and speed violations, were found to be significant [52,53]. Another study explored driving violations using driver behaviour questionnaires (DBQ) in Israel and found that factors like age, sex, and driving experiences directly influence the likelihood of committing driving violations [54]. Previously, literature relied primarily upon traditional statistical methods such as logistic regression [55] and canonical correlations [56], and Poisson/negative binomial regression [57,58]. Previous research also acknowledged the limitations of the statistical models while taking into account basic correlations between variables and results. Das et al. [55] have shown 62% accuracy in the possible fault assignment of drivers based on a logistic model. Alternately, a few studies have explored traditional machine learning methods like the random forest, gradient boosted decision tree (GBDT), and DTs for traffic violation taking into account risk for crashes with acceptable accuracy [39,59]. Additionally, a study analyzed taxi accidents in a survey to categorize the types of taxi drivers’ violations in China. The authors further analyzed the research data using simulations and found that accidents and driving violations happen due to driver behaviour, passengers, and vehicle performance [60].

With regard to what has been mentioned in the text of the literature, investigating the factors affecting the violation of urban taxi drivers is very valuable, since they form the world’s largest motorist’s community in metropolitan areas, and there is currently no particular analysis regarding their driving behaviour and their violations. Furthermore, variables related to the driver, the vehicle type, the road type, and location have not been fully understood regarding the taxi drivers. Variables, for example, weekends, weekdays, peak hours/off-peak hours, seasons, longitudes, latitudes, and types of violations regarding taxi, have not been evaluated in the previous studies. Therefore, this research is aimed at investigating the variables indicated for taxi driver violations.

## 3. Study Area and Data Collection

**Figure 1:**
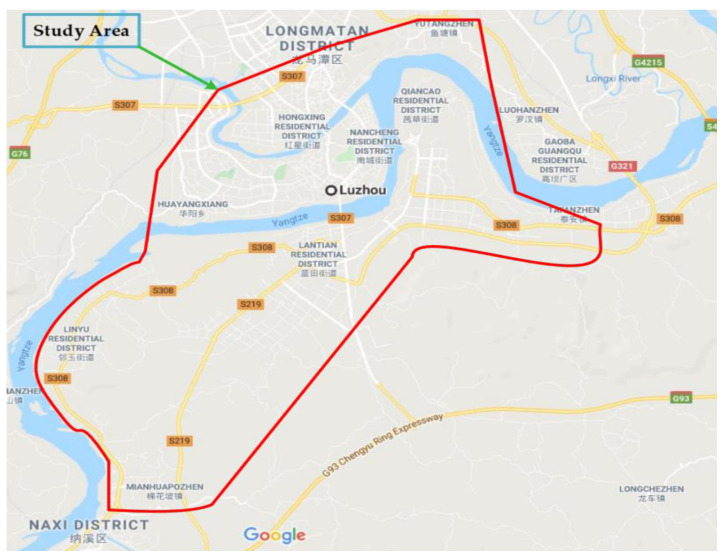
Study area (from Google map).

Luzhou is a prefecture-level municipality with an area of 12,246 km^2^, and a population over 1 million, located in the south-east of Sichuan Province, China. Located at the combination of the Tuo River and Yangtze River, the Luzhou port on the Yangtze River is the major port of Sichuan since the Chongqing Province in 1997. As per the National Bureau of the Statistics People’s Republic of China (PRC), by there of 2017, the country had 4.77 million paved roads and over 300 million registered vehicles [4]. The study area can be seen in Figure 1.

Traffic violation data for the year 2016 was obtained from the Sichuan traffic police department in the city of Luzhou. The data is extracted from off-site traffic monitoring and enforcement cameras that are installed at selected important locations. Thus, traffic violations along some short urban road segments may be missing. It is worth reporting that vehicle license plate numbers were not available in the dataset, which limits its application to record the violation history by specific vehicle/driver. This is a significant shortcoming that could be considered for forthcoming studies to give more useful insights about violation patterns by different vehicle/driver types.

## 4. Analysis of Descriptive Statistics and Violation Hotspots

In this study, traffic violations encountered by taxi drivers alone were considered since they constitute a significant proportion of total violations instances. A total of 64,156 violations by taxi drivers were used for the analysis after processing the data. Georeferenced violation data was collected on various attributes including detailed information on latitudes and longitudes, temporal attributes (such as peak/off-peak periods, weekdays/weekends, the season of the year), road types (as expressway, general urban highway, first/second class of highways) vehicle types, and resulting violation types. Table 1 presents the descriptive statistics for data utilized in this research.

**Figure 2:**
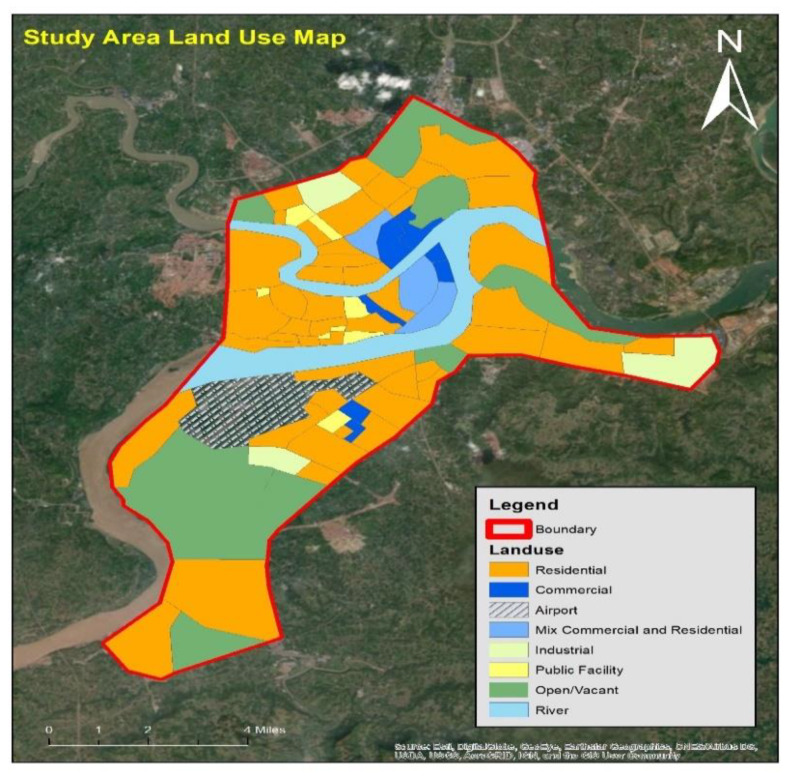
Land use map of study area.

In addition to descriptive statics, hotspot analysis for different violation types is also essential as they provide useful guidance to practitioners and decision-makers to identify sites where specific violation types are more prevailing. This, in turn, allows them to prioritize sites and propose suitable countermeasures to help alleviate the problem of non-compliance. To accomplish the hotspot analysis objective, the study area land use map (shown in Figure 2) was obtained from the city’s municipality. Information on land use is vital to establish a pattern of non-compliance among various city zones. The land use map shown below indicate that entire city area has been zoned into eight different land use i.e., residential, commercial, mixed commercial–residential, industrial, public facilities, open land, zone occupying and surrounding the city airport, and similar zone surrounding the river flow through the heart of the city. More than 60 percent of the area is pure residential with low to medium population density in the city’s suburbs and highly dense areas surrounding the downtown area. The commercial zones are mostly located in north-east directions.

**Figure 3:**
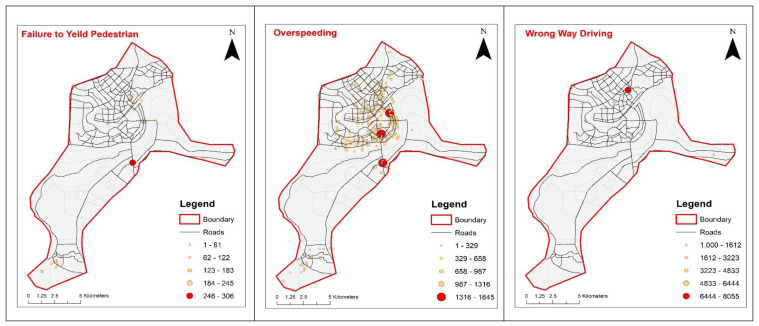

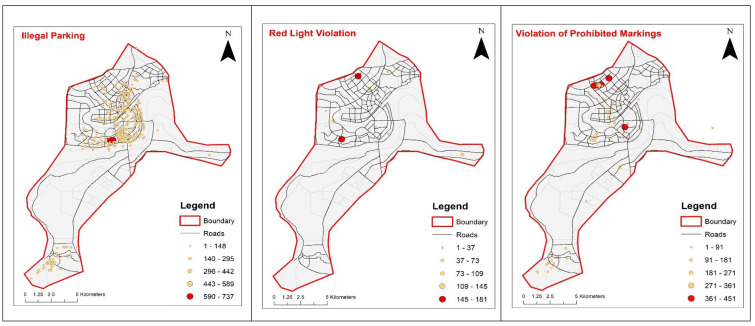
Distribution of hotspots by different violation types.

For identification of violation hotspots, an Arc map was used to determine the distribution of violations and to identify hotspots in the study area. The analysis was done using the nearest neighborhood. The method determines the spatial pattern of violations and the presence or absence of violations clusters. Based on the violation clusters, hotspots were identified. There were a total of seven types of traffic violations observed in the study area. Data for each violation type were extracted from shapefile using selection by attribute in the Geographic Information System (GIS). A separate shapefile was made for each violation type after the extraction of the data. GIS software used (version 10.3.1) for this study was obtained from its pioneer developers Environmental System Research Institute (ERSI) headquartered in Redlands, California. In recent years, the software has become quite popular for various applications in transportation such as identification of hotspots for urban travel patterns and accident analysis [61]. For making the data ready for hotspot analysis, each violation data point was integrated into a GIS in order to maintain the integrity of shared feature boundaries by making features coincident if they fall within the specified threshold tolerance. A spatial statistics tool (Collect Event) was used in order to convert the data to the weighted point. Hotspots for each violation type were categorized using equal intervals. Moreover, hotspots were divided into five categories, including very low, low, medium, high, and very high, based on the frequency of the violation. Figure 3 presents the hotspots map for the six most common violation types in the study area. Hotspot analysis of violation shown below indicate that incurred traffic violations are mainly located along both minor and major general urban roads. It is also worth noting from the same figure that a high concentration of violations was observed surrounding densely populated residential zones. In addition, it is clear from the figure that, although the spread and extent of violation hotspot maps for different violation types are different, they are mostly concentrated around the common epicenter. This observation is well-intuitive because the epicenters for the majority of observed violation types are located in the downtown or central business districts of the study area. These downtowns are the hub to diverse activities and thus host a significant proportion of violation hotspots.

Evaluating each hotspot independently, it is evident from Figure 3 that violation type “failure to compliance with a pedestrian crossing,” is concentrated in the city center mainly near the signalized intersections, which is intuitive. Similarly, some of the hotspots belonging to the same category were observed along the major road in the south of the study area. The road is going out of the city with decreased built-up areas. The drivers usually drive their vehicles in high speed and ignore pedestrians in the process, particularly in the absence of pedestrian traffic. For violation type, “illegal parking” had a total of two hotspots; both of them were very close to each other and located in a highly-populated mixed residential area with a high concentration of public facilities. These hotspots are dominant in a residential area because of two primary reasons. Firstly, residents come to these public facilities and do not find a place to park their vehicles. Secondly, this is a highly dense residential neighborhood where residents violate parking prohibition upon not finding parking spaces. Over-speeding is the most common traffic violation observed in the study area. A total of three hotspots were identified based on the violation data. Two hotspots were located in the city center, while the third was located on the major highway in the south of the city center. Over-speeding in the city center can be associated with human psychology to not getting stuck in the traffic. While highways usually see lesser traffic density, which offers more room for over-speeding.

Additionally, over-speeding compliance issues were frequent along routes with no or few speed cameras compared to those having strict speed regulations. Red-light violations was another major compliance issue observed in the study area. Two hotspots were identified for a red-light traffic violations. One in the industrial area, which is in the north of the city center, while the other one was in residential area in the west of city center. In central business districts (CBD) traffic is usually monitored through a traffic surveillance cameras and other surveillance equipment, which discourage red-light violations in these areas. Thus, a significant proportion of these violation types were reported where a strict traffic surveillance system was not in place or operational. Wrong-way driving was another violation type persistent in the study area with relatively low frequencies compared to the over-speeding and red-light violation. A large percentage of this violation type was concentrated in CBD, where a high volume of traffic and extreme congestion frustrates the drivers to use the wrong way and dedicated lanes in the hope of avoiding congestion and reach their destinations timely. Finally, prohibited road marking violations was another non-compliance problem prevailing in the city. A total of four hotspots were identified for this type of traffic violation. Three of them were located in the industrial area, where taxi drivers usually ignore the prohibited markings on the road. The violation of prohibited markings in the commercial area was associated with narrow roads and minimal opportunity for the driver to take care of these markings. Drivers in CBD mainly ignored the pavement markings and tended to use shoulders to escape heavy traffic. 

## 5. Traffic Violation Prediction Using Machine Learning (ML) Methods

Orange data mining toolbox in python was used to analyze the dataset with machine learning (ML) algorithms [62]. Orange version 3.24.1 downloaded and installed on a Dell with 8 GB of Random Memory Access (RAM), 3.79 GHz, a 64-bit Operating System (OS), and the integrated Radeon R7 Graphic Application Processing Unit (APU). The experiment was completed using an Advanced Micro Devices (AMD) R7-M460 Discrete/Hybrid. In addition, Orange toolbox in python contains a whole range of approaches to pre-processing and modelling of data. A brief overview of the classification algorithms used in this work are given in the following subsections.

### 5.1. Decisions Tree (DT)

**Figure 4:**
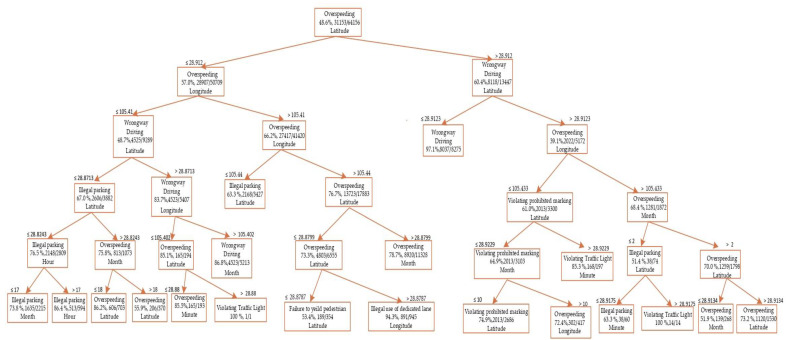
Prediction results of the decision tree method.

Decision tree (DT) has a powerful capability for the detecting trends in big data sets, and has a non-parametric data mining approach. The aim of using a decision tree is to develop a training model to predict the class or value of the target variable through the easy decision-making rules that can be derived from (previous) training data. Moreover, in DTs, we start from the root of the tree to predict a class label for a record. The root attribute values are further compared to the record’s attribute. Based on the comparison, we follow the branch of this value and jump to the next node. Decision trees classify the instances through sorting the tree from the root to some leaf or terminal node and by providing the instance classification with the leaf node. Every tree node serves as a test case for a particular attribute and the possible answers to the test case correlate with each edge descending from the node. This is a recursive method and repeated for all sub-trees that are rooted in the new node. In addition, the DT parameters include a minimum number of instances in leaves, splitting into smaller subset, maximum number of depths, and stopping the nodes from splitting once the required majority threshold has been reached. The findings are shown in Figure 4 as a tree viewer. The schematic for sequential decision-making during the decision tree algorithm for predicting specific violation type is shown in Figure 4. 

### 5.2. AdaBoost

AdaBoost, or adaptive bosting, is an ensemble boosting classifier introduced by Yoav Freund and Robert Schapire in 1996 [63]. AdaBoost utilizes various classifiers to improve the accuracy of classifiers and it is an iterative ensemble technique. AdaBoost classifier creates a strong classifier through the combination of several weak classifiers to ensure have a high accuracy. The key concept behind AdaBoost is to determine the weights (w) and to train the data sample (i) in each iteration (t) so that the strange or unusual results are predicted accurately. Initially, AdaBoost chooses a random subset of training set $D=(x_{i},y_{i})$ where by each $x_{i}$ example is the vector of an attribute value that belongs to a space X domain and every $y_{i}$ class label is related to $x_{i}$ which belongs to a Y and it trains the model of AdaBoost iteratively by choosing the training set based on the exact prediction of the last training. It allocates higher weight (w) to incorrect classified observations, so that these findings will have a high probability for classification in the next iteration. Moreover, in each iteration, it also assigns the weight to the trained classifier according to its accuracy. The more precise classifier will receive high weights. This method iterates once the complete training data matches without error or until reaching the maximum number of estimators specified. Additionally, the algorithm will iterate all possible features and estimate the error of each feature on each instance for each stage during each classifier training. The best feature is then selected as the first weak classifier. The weak learner’s job is to recognize a weak hypothesis $h_{t}:X\to \{-1,+1\}$, that is suitable for distribution $D_{t}$. The aim is to select $h_{t}$ to minimize error $\in_{t}$.

(1) $$\in_{t}=P_{r_{i∼D_{t}}}(h_{t}(x_{t})\neq y_{i})$$

(2) $$\alpha_{t}=\frac{1}{2}\ln(\frac{1-\in_{t}}{\in_{t}})$$

updating the distribution D and emphasizing the misclassified points, the final AdaBoost classifier formula are given in below Equation (3):

(3) $$H(x)=Sign(\sum_{i=1}^{T}\alpha_{t}h_{t}(x))$$

where $h_{t}$ is weak learner, $\alpha_{t}$ is coefficient, and H(x) is output the final hypothesis. AdaBoost predicts, similarly to random forest (RF), by adding several DTs to each sample and by combining predictions individual trees. Furthermore, instead of taking the average predictions in the forest by each DT or large proportion in the case of classification, while each DT in the AdaBoost algorithm adds a varying extent to the final prediction. In this study, we considered the base estimator as DT. Since our problem was multi-class, we used stagewise additive modelling with the multiclass exponential function algorithm (SMME) to improve the AdaBoost model. In addition, we used the linear loss function while prediction and classification of traffic violations.

### 5.3. Stack Model

Stacking is an ensemble learning technique combining many classifications or regression models through a meta-classifier or meta-regressor. It was introduced in 1992 by Wolpert [64]. In comparison to bagging and boosting, a stacking ensemble classifier does not use weighted or equivalent voting from sub classifiers to predict the output. The stacking method involves output created from the base level (level-0) classifiers as an input to meta level (level-1) classifier to enhance classification efficacy employing the cross-validation method. Moreover, it consists of a list of learners L with a specific set of parameters. In other words, firstly, the base level classifiers using the training dataset are trained. Afterward, the combiner models are then trained to make a final prediction, using all base level classifiers’ predictions as additional inputs. Given a dataset D of traffic violations with attributes $x_{i}$ associated with class labels $y_{i}$. Where, $D=(x_{i},y_{i}),\;i=1,2,\ldots ,n$ refers to Level-0 of the traffic violation dataset. Based on stratified K-fold cross validation D is divided into k different parts of $D_{1},D_{2},D_{3},\ldots \ldots ,D_{k}$. Let $D_{k}$ and $D^{\left(-k\right)}=D-D_{k}$ define is test and training set of kth fold cross validation. Further, J algorithms $B_{1},B_{2},\ldots ,B_{j}$ are applied to training part $D^{\left(-k\right)}$ to make J level-0 classifiers $C_{1},C_{2},\ldots ,C_{j}$. The prediction of each K-fold for $D_{k}$ of J level-0 classifiers with real class label are used to make meta level $\left(MD_{k}\right)$. It would be used when defining level-1 classification. By developing a complete metadata vector $\left(MD_{k}\right)$, also known as level-1 data obtained by the union of each $MD_{k}$, where k=1,…,K. In addition, we used the $B_{m}$ algorithm for the meta level classification of $C_{m}$ during the cross-validation process. The $B_{m}$ can be one of $B_{1},B_{2},\ldots ,B_{j}$ or a specific one during development of $C_{m}$.This technique allows the whole data to be trained with the learning algorithms $B_{1},B_{2},\ldots ,B_{j}$ to build final base level classifiers $C_{1},C_{2},\ldots ,C_{j}$ after formation of meta-level data. The algorithm of stacking model is given below as Algorithm 1.
**Algorithm 1. pseudo code of stack model**Input violations dataset,$D=((x_{1},y_{1}),(x_{2},y_{2}),\ldots \ldots ,(x_{n},y_{n}))$, $x_{n}$ represent attribute vector, n is number of observations, and where $y_{n}$ is for predictions or outcomes.
Level-0 classification models $C_{1},C_{2},\ldots ,C_{j}$Level-1 meta learner, $C_{m}$Ensemble size J,For j=1 to J$C_{j}$= creation of Level-0 models (D) (Creating Level-0 models from dataset)EndCreation of New dataset,$D_{new}=0$ For i=1 to n      For j=1 to JTo make prediction with meta learner or classifier$C_{ij}=C_{j}(x_{i})$ End$D_{new}=D_{new}\;\cup \;((C_{i1},C_{i2},\ldots \ldots \ldots ,C_{jn}),y_{n})$ (Combining with different classifiers)EndTraining meta classifier or Level-1 with new dataset$C_{m,trained}=C_{m}(D_{new})$EndOutcomes:Return final predictions from $C_{m,trained}$.

**Figure 5:**
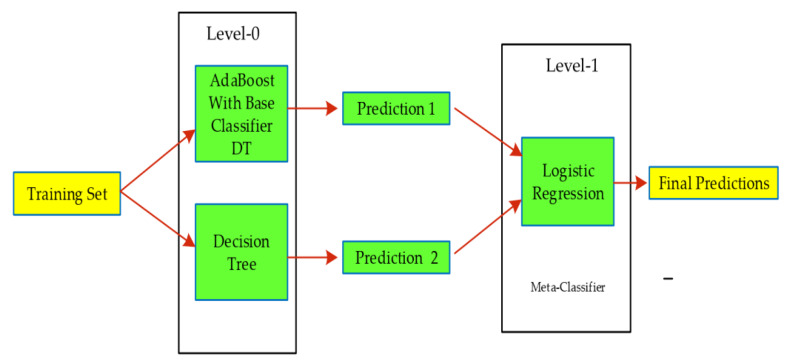
Schematic generalization of stacked framework.

For the current study, the stacking generalization technique was developed using two steps. The first step has a base level classifier that is DT and AdaBoost, while the meta level has a logistic regression that combines the base level classifier as an input to make final predictions. The applied stack model for this study can be seen in Figure 5.

### 5.4. Model’s Evaluation Metrics

In this analysis, we used the most common evaluation metrics to test the efficiency of the different classification models: precision, recall, accuracy, F-1 score, and confusion metrics. For the confusion matrix, samples were divided into four categories for classification problems: true (TP) positive, true negative (TN), false positive (FP), and false negatives (FN), and can be seen in below Table 2.

Precision quantifies the number of positive predictions that are made correctly while the recall quantifies the number of correct positive predictions that could have been made from all the positive predictions. The formula for calculating precision and recall could be found in Equations (4) and (5). The F-score comprises both the recall and the precision and calculated from Equation (6). Accuracy is the proportion of the correct sample to the total number of samples, and can be calculated from the Equation (7). Similarly specificity can be calculated from Equation (8):

(4) $$Precision=\frac{TP}{TP+FP}$$

(5) $$Recall=\frac{TP}{TP+FN}$$

(6) $$F-score=\frac{1}{Precision}+\frac{1}{Recall}$$

(7) $$Accuracy=\frac{TP+TN}{TP+TN+FP+FN}$$

(8) $$Specificity=\frac{TN}{TN+FP}$$

## 6. Results and Discussions

The model applied with base classifier DT and the parameters include the number of estimators and learning rate. The learning rate and number of estimators were strongly correlated with each other in order to fit the number of weak learners. To maintain a constant training error, smaller values of learning rate with larger number of weak learners is required. Empirical proof indicates that smaller learning rate values support better test errors [65]. The values obtained for learning rate and number of estimators were 1.0 and 70. The performance of the model via the confusion matrix, precision, and recall are shown in Table 3, and were achieved using stratified 10-fold cross validation. On the other hand, the accuracy of the DT model achieved 85%, when the values of parameters including a minimum number of instances in leaves, splitting into a smaller subset, and the maximum number of depth, were 1, 1, 1000. The number of nodes and leaves were 16,565 and 8283, respectively. The performance of model via confusion matrix, precision and recall are listed in Table 4 and were achieved using stratified 10-fold cross validation. In stacking, the logistic regression was used as aggregation, which provides a method to aggregate the input models like AdaBoost with DT estimator and DT. The accuracy achieved 86%, when meta-classifier logistic regression regularization type lasso (L-1) and strength was C = 1. The performance of the model via confusion matrix, precision, and recall are shown in Table 5 and were achieved using stratified 10-fold cross validation.

In reality, taxi drivers frequently commit to traffic violations, but in this study, we just focused on seven violations types. Among these violations, the most common type of violation happened on the general urban road due to the over-speeding of taxi drivers, which contributed to 48.56% of total violations. Moreover, illegal parking of taxi drivers contributed to 20.07%, wrong-way driving 19.07%, violation of prohibited road markings 6.51%, and illegal use of dedicated lane 2.15%. In addition, the study showed that the autumn season (September, October, and November) was more likely to have violations compared with the spring, summer, and winter seasons. It contributed to 30.47% of total violations, and over-speeding was one of the top violations, which happened in autumn as well as in other seasons. The second most common violation type that happened was illegal parking, which also occurred in autumn and contributed to 20.70% of total violations. Similarly, if we see in light of weekdays, most violations took place in working days instead of weekends. Approximately 75.54% 24.46% happened during weekdays and weekends, respectively.

Over-speeding violations occurred 36.28% during working days and 12.28 % on weekends. Likewise, illegal parking added 16.37% on working days, and 3.70% on weekends. Moreover, wrong-way driving added 14.36% on working days and 5.42% on weekend days. The distribution of violations in terms of peak hours (9:00 am and 10:00 am, 16:00 pm and 17:00 pm) and off-peak hours are stated in Table 1. In fact, violations that occurred in the morning peak hour were comparatively higher than peak hours in afternoon. In morning peak hours, the violation was 24.58%, while in afternoon peak hours the percentage was 23.8 %. As discussed above, over-speeding was the most contributing type of violation in all scenarios even from seasons to months or days to hours etc. Generally, most violations occurred from Monday to Wednesday compared with other days. It is notable that taxi drivers commit violation of over-speeding as it is more likely to be associated with urban roads as vehicles need fast driving on highways and freeways. Keep in mind this fact, on freeways, expressways, and urban motorways the number of violations from taxis was particularly low compared to general urban road or arterials. In addition, usually speed monitoring devices on expressways are better equipped. Drivers on expressway and motorways understand that if traffic regulations are violated here, there is a greater risk of being caught and a major penalty. Furthermore, expressway drivers are supposed to be long distance commuters who seem to be aware and therefore are more watchful about the possible dangers of traffic violations.

### 6.1. Model’s Comparison

**Figure 6:**
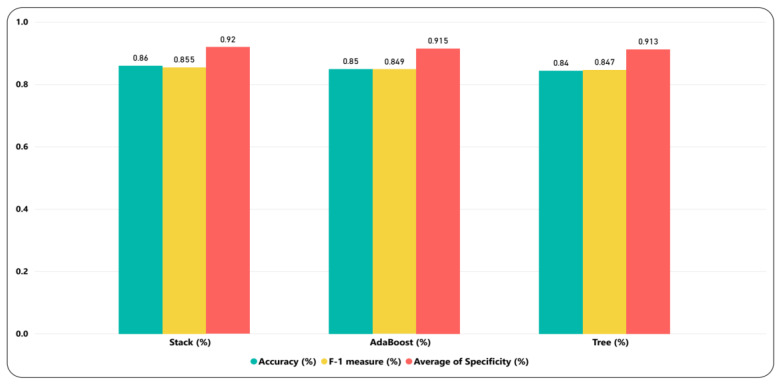
Accuracy, specificity, and F-1 score comparison for different models.

**Figure 7:**
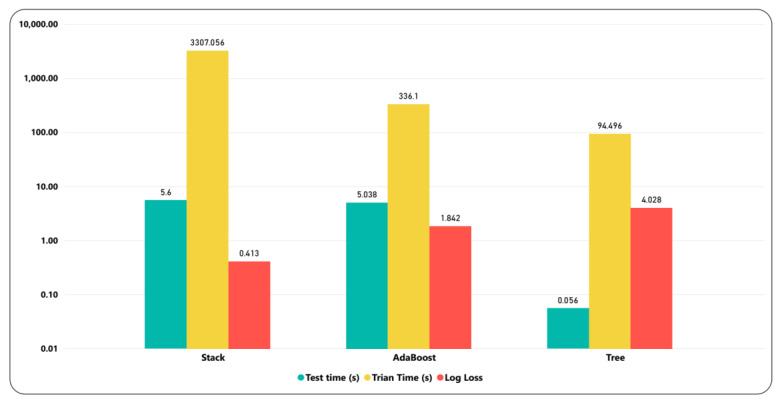
Runtime (seconds) with log loss comparison for different models.

The model comparison is made in order to see the efficacy of applied models. The model’s performance is checked in terms of accuracy, specificity, and F-1 score. Among these models, the stack model outperformed the base models, including DT and AdaBoost. Figure 6 shows the accuracy obtained for DT and AdaBoost was 0.842 and 0.848, respectively, while the obtained F-1 score for these models were 0.847 and 0.849, respectively. Moreover, the specificity for the stack, AdaBoost, and DT was 0.92, 0.915, and 0.913, respectively. The accuracy and F-1 score achieved for the stack model were 0.86 and 0.855, respectively. The stack model took more time while training and testing compared to the baseline models but obtained less log loss. Figure 7 shows the training, testing time (seconds), and log loss for all models. The log loss for the stack, AdaBoost, and DT was 0.413, 1.842, and 4.028, respectively. The log loss of stack model was lower comparing to AdaBoost and DT. Less log loss demonstrates higher accuracy, which further validated the performance of stack model. Among these model’s DT took less time to test and train compared to stack and AdaBoost models.

### 6.2. Proposed Mitigation Strategies

In this section, several potential mitigations strategies have been proposed to reduce the burden of traffic violations and to ensure smooth, safe, and efficient traffic operations in the study area. For red-light violations, appropriate countermeasures include installation of enforcement cameras at urban intersections, provision of signal ahead sign, installation of traverse rumble strips, activation of advance warning flashers, and improved pavement surface conditions. Previous studies indicate that most of these proposed countermeasures were very effective to limit red-light violations in urban metropolitan areas around the world [66,67]. Safety education and awareness programs could also significantly improve road safety situations [68] and should be practiced more frequently. In this regard, a comprehensive pedestrian awareness program and the establishment of pedestrian safety as a priority area must be taken to avoid pedestrian crossing violations. Various speed and traffic calming measures may be installed at violation hotpots to ensure the obeyance of traffic rules and regulations. For example, to discourage over-speeding along busy residential streets, a few mitigation measures that could be useful include the installation of speed cameras to arrest absconders, provision of speed humps, proactive traffic forecasting, and various other automated enforcement Intelligent Transport System (ITS) programs [69,70].

Similarly, the provision of adequate traffic control devices (traffic signs, markings, and channelization devices) and enhanced curve delineation should be introduced to improve miserable road safety situations. The advanced data recording system is also mandatory to conduct a thorough analysis, establish priorities, and to suggest appropriate mitigations guidelines for practitioners [71]. The current data recording system has missing information on several important variables (i.e., drivers’ socio-demographic attributes, vehicle license plates, features of surrounding built environment, etc.) that limit the use of data investigations to explore latent correlations among contributing factors. Finally, effective coordination and engagement among key stakeholders is extremely vital and must be ensured to facilitate safe and efficient traffic operations.

## 7. Conclusions

Risky and aggressive driving behaviour is a critical social and public health concern worldwide. Previous studies have mostly used statistical analysis methods to investigate violation contributing factors caused by such uncivilized driving attitudes. However, statistical methods have received widespread criticism regarding poor prediction performance, as well as their inferior ability to capture correlations among dependent variables. Hence, in this study we investigated patterns of traffic violations that occurred in the city of Luzhou, China using spatial analysis methods and different machine learning algorithms. Georeferenced traffic violation data for the year 2016 was obtained from local police departments. Violations committed by taxi drivers were extracted for analysis, since they account for a significant proportion of total violations reported in the study area. During the first phase of the study, a detailed descriptive analysis of the data was conducted that revealed distribution of violations based on different variables. Over-speeding had the highest proportion of 48.56% of total violations followed by illegal parking 20.07%, and wrong-way driving 19.77% occurring in the study area. This was followed by developing hotspots in Arc GIS for different violation types along with rational discussions of each with adjacent land use. Violation hotspots were mostly concentrated in the CBD along densely populated and congested links, whereas over-speeding violations were observed during off-peak periods and mostly along expressways. Finally, during the third phase, classification and prediction of various violation types observed were accomplished using three state of the art machine learning (ML) models i.e., AdaBoost, DT, and stacking. The stack model prediction performance indicates good efficacy with the accuracy of 86% in predicting the traffic violations. Moreover, the stack model outperformed AdaBoost and DT. The model comparison showed improved predictive performance of the stack model in terms of log loss, specificity, and a slightly higher test time compared with the DT and AdaBoost models, which further validates the efficacy of the proposed approach.

The findings of this study could provide essential guidance for decision makers to initiate concrete steps for engineering applications in road safety management. This study has few limitations that could be investigated in future research. In forthcoming studies, it would be interesting to comprehensively explore the impact of driving styles, working time, and regular travel on realistic traffic violations of taxi drivers. Furthermore, studies could focus on exploring the influence of detailed socio-demographic characteristics of drivers, which were unfortunately not available for this study. Finally, the present analysis was restricted to only one city, therefore the inferences made could be better assessed by extended it to other cities.

## Figures and Tables

**Table 1:** Descriptive statistics of the violation samples (*N* = 64,156).

Type of Violation	Count of Type of Violation	Percentage of Total Violation	Variable Type
Over-speeding	31,153	48.56%	Response
Illegal parking	12,878	20.07%	Response
Wrong way driving	12,687	19.77%	Response
Violation of Prohibited road markings	4174	6.51%	Response
Illegal use of dedicated lane	1381	2.15%	Response
Failure to yield pedestrian	1245	1.94%	Response
Violation of traffic signals/lights	638	0.99%	Response
**Location**			
Longitude	64,156	-	Predictor
Latitude	64,156	-	
**Hours of the day**			
Peak hours (9:00 am–10:00 am, 15:00 pm–16:00 pm)	33,137	51.65%	Predictor
Off-peak hours (11:00 am–14:00 pm, 17:00 pm–8:00 am)	31,019	48.35%	
**Season**			
Autumn	19,546	30.47%	Predictor
Summer	17,760	27.68%	
Winter	14,863	23.17%	
Spring	11,987	18.68%	
**Week**			
Working day	48,461	75.54%	Predictor
Weekend	15,692	24.46%	

**Table 2:** Confusion matrix for evaluating model’s performance.

Actual Condition	Predicted Condition	
	Positive	Negative
**Positive**	True Positives (TP)	True Negatives (TN)
**Negative**	False positives (FP)	False Negatives (FN)

**Table 3:** Confusion matrix for AdaBoost model.

Actual				Predicted				
	Pedestrian	Illegal Parking	Illegal Use of Dedicated Lane	Over-Speeding	Prohibited Markings Violation	Signal Violation	Wrong-Way Driving	Precision
Pedestrian	71.7%	1.1%	0.1%	0.8%	0.2%	0.2%	0.0%	71.7%
Illegal parking	8.9%	72.5%	0.0%	12.0%	1.9%	0.2%	0.3%	72.5%
Illegal use of dedicated lane	0.2%	0.1%	99.6%	0.0%	0.0%	0.0%	0.0%	99.6%
Over-speeding	18.7%	25.2%	0.1%	85.4%	7.7%	0.8%	1.4%	85.4%
Prohibited markings violation	0.4%	0.9%	0.1%	1.1%	88.0%	2.8%	0.4%	88.0%
Signal violation	0.1%	0.1%	0.0%	0.1%	0.7%	60.6%	2.0%	60.6%
Wrong-way driving	0.0%	0.3%	0.0%	0.6%	1.4%	35.4%	95.9%	95.9%
**Recall**	67.5%	68.5%	98.4%	87.8%	86.9%	48.0%	96.4%	

**Table 4:** Confusion matrix for Decision Tree (DT) model.

Actual				Predicted				
	Pedestrian	Illegal Parking	Illegal Use of Dedicated Lane	Over-Speeding	Prohibited Markings Violation	Signal Violation	Wrong-Way Driving	Precision
Pedestrian	69.6%	0.9%	0.1%	0.7%	0.2%	0.4%	0.0%	69.6%
Illegal parking	8.9%	70.3%	0.0%	11.8%	2.3%	0.6%	0.3%	70.9%
Illegal use of dedicated lane	0.2%	0.0%	99.6%	0.0%	0.0%	0.0%	0.0%	99.6%
Over-speeding	19.2%	27.8%	0.1%	86.4%	8.5%	1.2%	1.9%	86.4%
Prohibited markings violation	0.8%	0.7%	0.2%	1.0%	86.7%	3.2%	0.4%	86.7%
Signal violation	0.2%	0.0%	0.0%	0.0%	0.8%	62.4%	2.1%	62.4%
Wrong-way driving	0.0%	0.1%	0.0%	0.1%	1.5%	35.4%	95.3%	95.3%
**Recall**	79.0%	69.6%	99.3%	85.9%	88.3%	49.2%	97.9%	

**Table 5:** Confusion matrix for stack model.

Actual				Predicted				
	Pedestrian	Illegal Parking	Illegal Use of Dedicated Lane	Over-Speeding	Prohibited Road Markings	Signal Violation	Wrong-Way Driving	Precision
Pedestrian	74.1%	1.1%	0.1%	0.9%	0.2%	0.5%	0.0%	74.1%
Illegal Parking	8.9%	75.8%	0.0%	12.6%	2.1%	0.5%	0.3%	75.8%
Illegal use of dedicated lane	0.2%	0.0%	99.6%	0.0%	0.0%	0.0%	0.0%	99.6%
Over-speeding	19.2%	27.8%	0.1%	85.3%	7.5%	1.2%	1.9%	85.3%
Prohibited road markings	0.8%	0.7%	0.2%	1.0%	87.9%	3.2%	0.4%	87.9%
Signal violation	0.1%	0.0%	0.0%	0.0%	0.8%	68.4%	2.3%	68.4%
Wrong-way driving	0.0%	0.2%	0.0%	0.1%	1.5%	26%	95.2%	95.2%
**Recall**	64.7%	66.3%	99.3%	89.6%	87.4%	46.2%	98.3%	
